# *In vitro* neuroprotective potential of four medicinal plants against rotenone-induced toxicity in SH-SY5Y neuroblastoma cells

**DOI:** 10.1186/1472-6882-13-353

**Published:** 2013-12-12

**Authors:** Keabetswe Seoposengwe, Jacob John van Tonder, Vanessa Steenkamp

**Affiliations:** 1 Department of Pharmacology, Faculty of Health Sciences, University of Pretoria, Private Bag X323, Arcadia, Pretoria, South Africa

**Keywords:** Caspase-3, *Crinum bulbispermum*, Glutathione, *Lannea schweinfurthii*, Mitochondrial membrane potential, Neuroprotection, Reactive oxygen species (ROS), Rotenone, *Scadoxus puniceus*, *Zanthoxylum capense*

## Abstract

**Background:**

*Lannea schweinfurthii, Zanthoxylum capense, Scadoxus puniceus* and *Crinum bulbispermum* are used traditionally to treat neurological disorders. The aim of this study was to evaluate the cytoprotective potential of the four plants, after induction of toxicity using rotenone, in SH-SY5Y neuroblastoma cells.

**Methods:**

Cytotoxicity of the plant extracts and rotenone was assessed using the sulforhodamine B (SRB) assay. Fluorometry was used to measure intracellular redox state (reactive oxygen species (ROS) and intracellular glutathione content), mitochondrial membrane potential (MMP) and caspase-3 activity, as a marker of apoptotic cell death.

**Results:**

Of the tested plants, the methanol extract of *Z. capense* was the least cytotoxic; LC_50_ 121.3 ± 6.97 μg/ml, while *S. puniceus* methanol extract was the most cytotoxic; LC_50_ 20.75 ± 1.47 μg/ml. Rotenone reduced intracellular ROS levels after 24 h exposure. Pre-treating cells with *S. puniceus* and *C. bulbispermum* extracts reversed the effects of rotenone on intracellular ROS levels. Rotenone exposure also decreased intracellular glutathione levels, which was counteracted by pre-treatment with any one of the extracts. MMP was reduced by rotenone, which was neutralized by pre-treatment with *C. bulbispermum* ethyl acetate extract. All extracts inhibited rotenone-induced activation of caspase-3.

**Conclusion:**

The studied plants demonstrated anti-apoptotic activity and restored intracellular glutathione content following rotenone treatment, suggesting that they may possess neuroprotective properties.

## Background

Parkinson’s disease (PD) is the second most common progressive age-related neurological disorder. It is estimated to affect more than 6 million people worldwide [1]. The prevalence of the disease is reported to be 1-2% amongst those who are over 65 years and about 4% amongst individuals who are older than 80 years [2,3]. The disease is characterized by a marked selective degeneration and loss of dopaminergic neurons in the brainstem region, specifically the substantia nigra pars compacta, causing impaired dopamine signaling [4].

In order to investigate PD, use is made of an *in vitro* neuronal cell model. In the present study SH-SY5Y neuroblastoma cells were used since these cells share various biochemical and functional characteristics of innate neurons [5]. These include: the expression of dopamine and noradrenaline producing enzymes, acetylcholine, norepinephrine and various growth factor receptors [6]. Neurotoxins, such as environmental pesticides and herbicides that produce PD-like symptoms *in vivo*, are commonly used to study PD in different neuronal cell lines [7,8]. It is thought that the pesticide, rotenone, induces PD-like symptoms in neurons through disrupting adenosine triphosphate (ATP) supply [9]. This happens when rotenone forms a complex with members of the mitochondrial electron transport chain (ETC), specifically at complex I, resulting in limited ATP production [10].

Presently, there is no cure for PD [11]. Existing therapies are aimed at increasing CNS dopamine levels, enhancing dopaminergic cell survival and modifying clinical PD symptoms [12,13]. Unfortunately, most of these therapies have adverse effects [14,15], which has resulted in extensive research into complementary and alternative medicines that may be of benefit [16,17]. In the United States, United Kingdom and Korea, approximately 40%, 39% and 76% of PD patients, respectively, have admitted to self-medicating in the form of herbal remedies [16,18].

Four herbal remedies which are either traditionally reported to be used in the treatment of neurological disorders or which are implicated in neurological disorders were selected for investigation. Infusions of the roots of *Lannea schweinfurthii* (Anacardiaceae) are reported to enhance memory and are used as a sedative [19,20] whereas decoctions of the roots of *Zanthoxylum capense* (Rutaceae) are used to treat epilepsy [21]. The bulbs of *Crinum bulbispermum* and *Scadoxus puniceus* (Amaryllidaceae) are reported to possess anticonvulsant activity and cause CNS excitation and depression, respectively [22,23]. The aim of this study was to investigate the cytoprotective potential of these four herbal remedies against rotenone-induced toxicity in SH-SY5Y neuroblastoma cells.

## Methods

### Plant specimens and preparation of extracts

The plants investigated in this study were either a gift from the South African National Botanical Institute (SANBI, Tshwane) or collected by an expert botanist in Venda, Limpopo. Voucher specimens of the plants are deposited at the SANBI herbarium or in the Department of Toxicology (Onderstepoort Veterinary Institute, Pretoria).The plants investigated included: the root-bark of *Lannea schweinfurthii* (Engl.) Engl. (voucher LT 19), roots of *Zanthoxylum capense* Thunb. Harv (voucher LT 4), and the bulbs of *Scadoxuspuniceus* (L.) Friis & Nordal (voucher SANBI) and *Crinum bulbispermum* (Burm. f.) Milne-Redh.&Schweick (voucher SANBI).

Plant material was air-dried and ground to a fine powder using a Wiley Mill (Model no 2, Philadelphia U.S.A). Ground plant material (1.5 g) was extracted with 15 ml of either methanol or ethyl acetate. Extracts were incubated at room temperature in an ultrasonic bath for 30 min and then placed on an orbital shaker for a further 2 h, after which they were incubated at 4°C for approximately 20 h. To separate debris, extracts were centrifuged at 1000 × *g* for 10 min, syringe-filtered (0.22 μM) and dried under reduced pressure using a rotary vacuum evaporator (BUCHI Rotavapor R-200, LABOTEC). Dried extracts were reconstituted in dimethylsulfoxide (DMSO) and aliquots stored at −20°C until use. Yields were: *L. schweinfurthii* 4.0% and 4.3%; *Z. capense* 18.3% and 4.2%; *S. puniceus* 8.7% and 4.5% and *C. bulbispermum* 3.7% and 14.0% for the methanol and ethyl acetate extracts, respectively.

### Cell culture maintenance and harvesting

SH-SY5Y neuroblastoma cells (ATCC no. CRL-2266) were purchased from the American Type Culture Collection and cultured in 75 cm^3^ culture flasks at 37°C under an atmosphere of 5% CO_2_ and humidified air. Cells were grown in Ham’s F12 medium, supplemented with 10% heat-inactivated fetal calf serum (FCS) and 1% penicillin-streptomycin. The medium was replaced every 2-3 days, as required. Once cells reached a confluency of ≈ 80%, medium was discarded and cells were washed with phosphate buffered saline (PBS). Cells were detached using a 0.125% Trypsin/Versene solution and harvested by centrifugation at 200 × *g* for 5 min. Cells were resuspended in 1 ml of medium and viable cells were counted using trypan blue (0.4% w/v in PBS).

### Cytotoxicity of the individual test compounds and effects of the plant extracts on rotenone-induced cytotoxicity

Cytotoxicity was determined using the sulforhodamine B assay (SRB) as described by Vichai and Kirtikara [24]. Cells (100 μL, 1 × 10^5^ cells/ml) were pre-seeded into 96-well plates followed by the addition of 80 μL of 2% FCS-supplemented medium and incubated for 24 h. To determine the cytotoxicity profiles of each plant extract or rotenone alone, a volume of 20 μL of either plant extract (final exposure concentrations of 0.78-100 μg/ml) or rotenone (final exposure concentrations of 0.128 nM-50 μM) was added to the plates and incubated for 72 h, after which the SRB assay was performed. Vehicle controls were exposed to 0.05% (v/v) DMSO in culture medium and wells containing culture medium only served as blanks. To assess the effects of the plant extracts on rotenone-induced cytotoxicity, cells were pre-treated for one hour with four non-toxic concentrations of each of the plant extracts prior to being exposed to rotenone for 72 h at concentrations of 10 nM, 50 nM and 100 nM. Minocycline (10 μM), which is known to counteract rotenone toxicity [25], was used as treatment positive control throughout the study. All subsequent experiments evaluating mechanistic parameters utilized a rotenone concentration of 50 nM.

The SRB assay was performed as follows: After the 72 h exposure period, 100 μL of the supernatant was aspirated from the wells and replaced with 100 μL of cold trichloroacetic acid (TCA) solution (30% w/v). Each plate was then incubated at 4°C for 1 h to fix cells to the plate. After incubation, the plate was gently washed with water (four times) to remove excess TCA. The plate was dried in a low-temperature oven, after which 100 μL of 0.057% (w/v) SRB solution was added to wells to stain the cellular protein contents. The plate was incubated for 30 min at 4°C and washed twice with 200 μL of a 1% acetic acid solution (v/v) to remove excess unbound dye. The plate was allowed to dry, after which the bound dye was dissociated using 200 μL of a 10 mM Tris base solution (pH 10.5). Absorbance was measured at 540 nm with a reference wavelength of 630 nm, using a Biotek EL_x_ 800_UV_ Universal plate reader.

Preliminary experiments indicated that rotenone induced approximately 50% cell death at a concentration of 50 nM. For this reason rotenone was used at a concentration of 50 nM for all subsequent mechanistic studies.

### Intracellular reactive oxygen species

Intracellular ROS production was assessed using the method described by Shaykhalishahi *et al.*[26], with slight modifications. Briefly, following 24 h exposure to the test compounds, 20 μL of 2’,7’-dichlorodihydrofluorescein diacetate (H_2_DCF-DA; 20 μM) in PBS solution was added to the relevant wells and the plates incubated for 30 min at 37ºC. Plates were then washed once with 100 μL of PBS to remove excess H_2_DCF-DA solution. An additional 100 μL of PBS was added to each well and the fluorescence intensity measured using a BMG Fluostar Optima fluorescent plate reader set at excitation and emission wavelengths of 492 nm and 525 nm, respectively. The values are expressed as the mean absorbance normalized to a percentage of the untreated control value. An established ROS-inducing agent, 2,2’-azobis-2-methyl-propanimidamide dihydrochloride (AAPH) (150 μM), was included as an additional positive control to ensure that the assay produced expected results.

### Intracellular glutathione levels

Intracellular glutathione levels were determined using the method of Nair *et al.*[27]. Following 24 h exposure to the relevant treatment, 20 μL of monochlorobimane (40 μM) in PBS solution was added to all the wells. The plates were incubated for 2 h at 37°C, followed by a 100 μL PBS washing step. After the addition of 100 μl of PBS, fluorescence intensity was recorded using a BMG Fluostar Optima set at excitation and emission wavelengths of 360 nm and 460 nm, respectively. The glutathione depleting agent, *N*-ethylmaleimide (NEM) (10 μM), was used as an additional positive control to ensure that the assay produced expected results.

### Mitochondrial membrane potential

Mitochondrial membrane potential (MMP) was determined according to Sternfeld *et al.*[28] with minor modifications. Briefly, after treatment of cells for 24 h, 100 μL of the supernatant was discarded and 20 μL of 5,5’,6,6’-tetrachloro-1,1’,3,3’-tetraethylbenzimidazolcarbocyanine iodide (JC-1; 10 μM) in PBS was added before plates were incubated for 30 min at 37°C and 5% CO_2_ in the dark. Excess dye was washed off using 100 μL PBS and an additional 100 μL PBS was added for fluorescence measurements. Fluorescence intensity was measured using a BMG Fluostar Optima fluorescence microplate reader set at excitation wavelengths of 492 nm and 520 nm, and emission wavelengths of 544 nm and 590 nm for the monomeric and aggregate forms of JC-1, respectively. The ratio of the fluorescence intensities at 590 nm (J-aggregates) / 520 nm (J-monomers) was used as an indication of MMP. The mitochondrial uncoupler, valinomycin (20 μM), was used as an additional positive control to ensure that the assay produced expected results.

### Apoptosis

The assay was conducted according to the method of van Tonder [29]. Briefly, following exposure, cells were lysed with 25 μL of a cold lysis buffer [10 mM 4-(2-hydroxyethyl)-1-piperazineethanesulfonic acid (HEPES), 2 mM ethylenediaminetetraacetic acid (EDTA), 5 mM3-[(3-cholamidopropyl)dimethylammonio]-1propanesulfonate (CHAPS), 5 mM beta-mercaptoethanol, 0.5 mM phenylmethylsulfonyl fluoride (PMSF)] and kept on ice for 30 min. Thereafter, 100 μL of reaction buffer (20 mM HEPES, 2 mM EDTA, 5 mM β-mercaptoethanol, 0.5 mM PMSF, 10 μM of a 7-amino-4-methylcoumarin-coupled caspase-3 substrate) was added to wells followed by an overnight incubation at 37ºC. Fluorescence intensity was measured using a BMG Fluostar Optima set at excitation and emission wavelengths of 360 nm and 460 nm, respectively. Staurosporine (11 μM), a general apoptosis inducer, was used as positive control to ensure that the assay produced expected results.

### Statistical analyses

For cell viability, the concentration that produces 50% cell death (LC_50_) was calculated by fitting a four-parameter Hill equation to the observed results. Two constraints (top = 100; bottom = 0) and a variable slope were used for fitting the non-linear model. Calculated LC_50_ values are expressed as the mean ± the standard error of the mean (SEM).

All experiments were carried out in triplicate on three separate occasions. Background signals (blanks) were deducted in all experiments. Depending on the normality of the data, either Mann–Whitney or Student’s t-tests were performed to test for significant differences between the means of the various groups. Results for endpoints assays were normalized to a percentage of the mean of vehicle controls and are presented as mean ± SEM. Significant differences from vehicle controls are indicated by ■ for *p* value < 0.05. Significant differences between treatment groups and rotenone treatment alone are indicated by * for *p* value < 0.05. GraphPad Prism 5.0 was used for all statistical manipulations.

## Results and discussion

The aim of the study was to determine the effects of methanol and ethyl acetate extracts of four South African medicinal plants on rotenone-induced neuronal toxicity using the SH-SY5Y neuroblastoma cell line. *In vitro* assays were employed to assess cytotoxicity, intracellular redox state (ROS and intracellular glutathione content), MMP and caspase-3 activity, of the plant extracts. These parameters were selected as oxidative stress and mitochondrial dysfunction appear to play key roles in PD manifestation [1]. Oxidative stress is the result of excessive free radical levels that originate from dopamine metabolism and electron transfer in the electron transport chain during energy metabolism [30]. Excessive free radicals deplete innate antioxidant defences [31]. When this happens, mitochondrial processes are affected causing mitochondrial dysfunction and thus disruption in brain function [32]. Caspase-3 activation executes apoptosis [33]. The effectors of apoptosis are responsible for the breakdown of the cellular cytoskeleton, mitochondrial DNA and DNA-associated proteins leading to neuronal cell death via mitochondrial-mediated apoptotic pathways [34].

### Cytotoxicity

#### SH-SY5Y viability following exposure to rotenone

From an initial dose-finding pilot study rotenone was observed to be the most cytotoxic of all the test compounds with a calculated LC_50_ value of 112 ± 1.05 nM. A second pilot study was conducted to test whether this calculated concentration actually produced the predicted 50% cell death. It was found that 50 nM of rotenone reduced viability to 48%, when compared to the vehicle controls. Results are in line with literature as rotenone, a classic complex I inhibitor, is known to be toxic to several cell lines [35]. Exposure to 0.1, 1 and 10 μM of rotenone for 4 h has been reported to decrease the viability of Neuro-2a mouse neuroblastoma cells by 86.78% ± 7.14%, 64.49% ± 3.41% and 50.11% ± 3.20%, respectively [36]. Another study, which was carried out using SK-N-MC human neuroblastoma cells, reported toxicity when cells were exposed to concentrations ranging from 10 nM to 1 μM of rotenone for 24–48 h [9]. Results from the present study and those reported in literature clearly demonstrate that the toxicity of rotenone is dependent on the concentration of rotenone and the duration of exposure.

#### SH-SY5Y viability following exposure to the individual plant extracts

The methanol extract of *L. schweinfurthii* produced a calculated LC_50_ value of 78.87 ± 2.10 μg/ml, while its ethyl acetate counterpart produced a calculated LC_50_ value of 36.02 ± 0.79 μg/ml. A study using a 20% aqueous-ethanol extracts of *Lannea stuhlmanii* reported no significant toxicity in human cervical carcinoma, human colon adenocarcinoma or human skin carcinoma cells, with > 85% viability after 72 h exposure to concentrations as high as 100 μg/ml [37]. Differences in cell type and/or plant species may account for this discrepancy.

Exposure to *Z. capense* extracts produced calculated LC_50_ values of 121.3 ± 6.97 μg/ml and 90.18 ± 0.56 μg/ml for the methanol and ethyl acetate extracts, respectively. Although the genus *Zanthoxylum* has been reported to possess cytotoxic properties, the isolated compounds alone have been reported to have negligible or no cytotoxic effects [38].

*S. puniceus* methanol extract had the lowest LC_50_ value (20.75 ± 1.47 μg/ml) of the plant extracts tested, indicative of a high toxic potential. The ethyl acetate extracts of this plant had an LC_50_ value of 37.40 ± 0.82 μg/ml. The ethyl acetate extract of *C. bulbispermum* was the least cytotoxic with an LC_50_ value of > 100 μg/ml. Contrary to this, the methanol extract produced an LC_50_ value of 46.18 ± 0.91 μg/ml. Both species are well-known for their high alkaloidal content [39], and the cytotoxic effects observed may be attributed to the presence of these compounds.

#### Inhibition of rotenone-induced SH-SY5Y cytotoxicity

**Figure 1:**
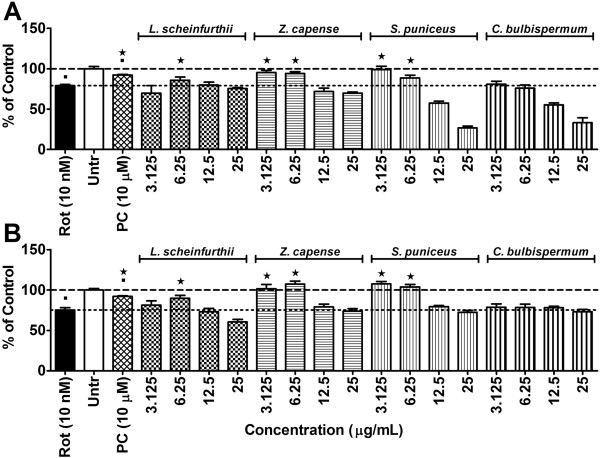
**Cytotoxicity results.** Effect of 1 h of pre-treatment with either **A)** methanol or **B)** ethyl acetate plant extracts on the survival of SH-SY5Y cells after 72 h exposure to 10 nM rotenone using the sulforhodamine B assay. Significant differences from the rotenone control are indicated by* representing p value < 0.05, while significant differences from vehicle controls are indicated by ■, also representing p value < 0.05. PC = positive control (minocycline at 10 μM); Rot = rotenone; Untr = Vehicle controls. (The dashed-line indicates 100% viability of the vehicle controls while the dotted-line is indicative of the level of cell death induced by the respective rotenone concentrations).

To determine the effects of the various plant extracts on rotenone-induced cytotoxicity (10 nM) the SH-SY5Y cells were pre-treated with selected sub-toxic concentrations of the plant extracts (3.125, 6.25, 12.5 and 25 μg/ml) for 1 h. The results are graphically presented in Figure 1A and B for the methanol and ethyl acetate extracts, respectively. The treatment positive control, minocycline, was observed to counteract rotenone toxicity by maintaining cell viability at > 90%. Minocycline is a lipophilic tetracycline antibiotic that is known to possess anti-inflammatory and antioxidant activities, besides its anti-bacterial activity [40-42].

With regards to the medicinal plants, both the methanol and ethyl acetate extracts of *Z. capense*, *S. puniceus* and *L. schweinfurthii* demonstrated cytoprotective properties in cells exposed to concentrations of 10 nM of rotenone (Figure 1). However, these same extracts did not show any significant cytoprotective effects at rotenone concentrations of 50 nM and 100 nM (data not shown).

### Cellular redox state

#### Intracellular reactive oxygen species

**Figure 2:**
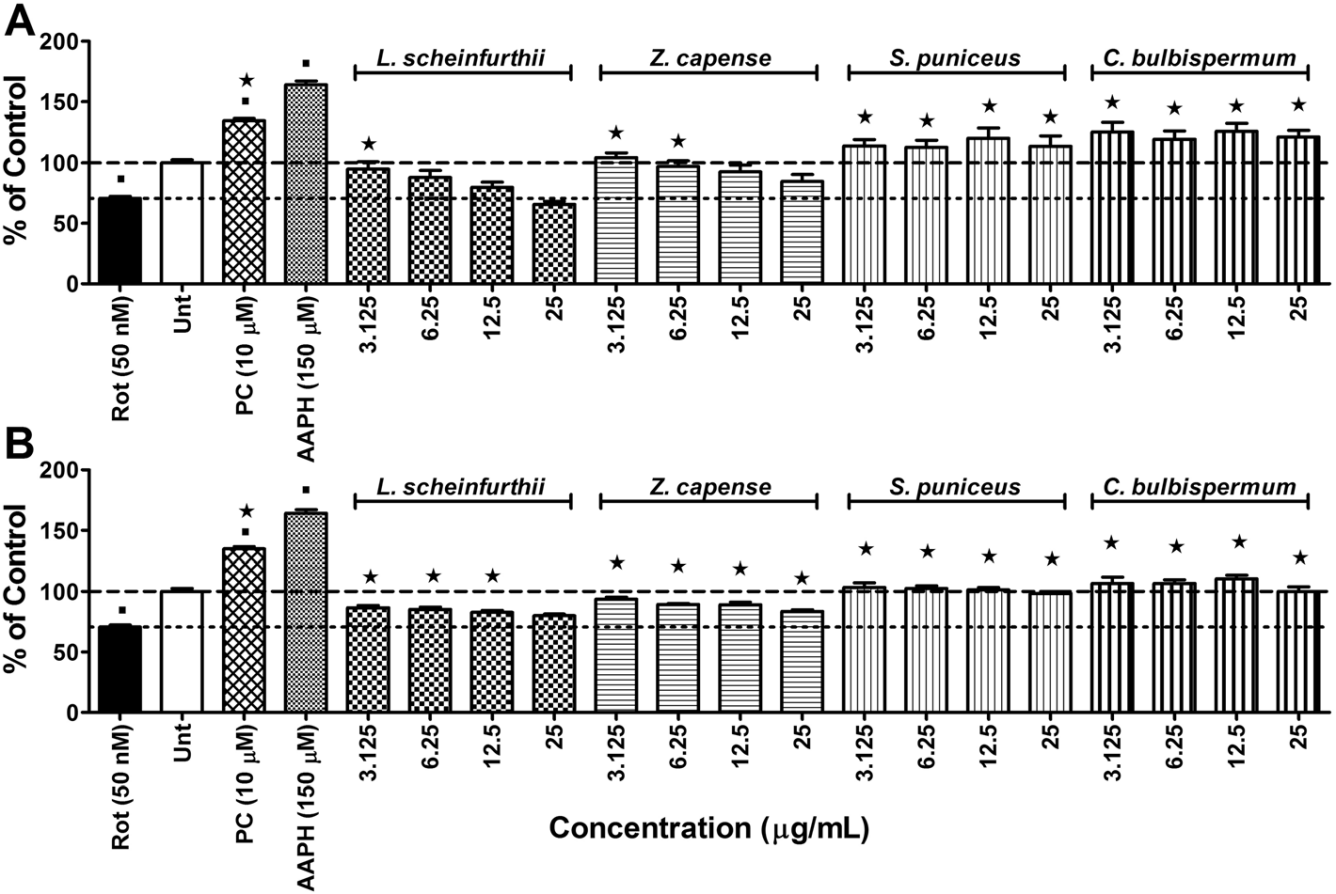
**Effects on radical generation.** Intracellular ROS levels in SH-SY5Y cells exposed to rotenone at 50 nM after 1 h pre-treatment with either A) methanol or B) ethyl acetate plant extracts following 24 h exposure period. Significant differences from the rotenone are indicated by * representing p value < 0.05, while significant differences from vehicle controls are indicated by ■, also representing p value < 0.05. PC = positive control (minocycline at 10 μM); Rot = rotenone; Untr = Vehicle controls. (The dashed-line indicates ROS levels in the vehicle controls while the dotted-line is indicative of ROS generation exposed to rotenone alone.

Significant (*p <* 0.05) intracellular ROS production was observed in cells exposed to the assay positive control, AAPH, indicating that the assay produced expected results (Figure 2). Compared to vehicle controls, there was no intracellular ROS production in cells exposed to 50 nM of rotenone for 24 h. Instead rotenone exposure significantly (*p <* 0.05) decreased intracellular levels of ROS. To confirm that rotenone exposure did not induce any intracellular ROS generation, experiments were repeated over a longer exposure period of 72 h. Still no significant intracellular ROS production was observed in cells exposed to rotenone alone, compared to vehicle controls (results not shown). The mitochondrial ETC complex I is the main site of ROS production from mitochondria [43] and ROS production can be enhanced by a defective mitochondrial ETC complex I [44]. This concept is in line with the report of Molina-Jimenez *et al*. [45] where rotenone induced ROS production in SH-SY5Y cells after 16 h treatment with 5 μM of rotenone, which stands in contrast to observations from the present study. It is possible that the rotenone concentration used in the present study (50 nM) might have been too dilute to achieve similar results, regardless of the exposure time. Results similar to that of the present study are reported from a study by Vrablic *et al.*[46]conducted on hepatocytes, in which a reduction in ROS production was observed after exposure to 50 nM of rotenone. Contrary to this, Gao *et al.*[47] have shown ROS production to be induced by 5 nM and 10 nM of rotenone after only 30 min of exposure in primary mesencephalic neuron glial cultures. It would appear as if rotenone-induced ROS generation *in vitro* depends on the type of cell line used, concentration of rotenone used and time of exposure to rotenone [48].

Minocycline, at a concentration of 10 μM, was able to counteract the effects of rotenone on intracellular ROS levels after 24 h of exposure. Not only did minocycline counter the effect of rotenone, it actually caused a significant (*p <* 0.05) increase in intracellular ROS levels, when compared to the vehicle controls (Figure 2). Observations from the present study contradict the notion that ROS generation plays a central role in the mechanism of rotenone-induced cytotoxicity as 50% cell death was observed without any significant increase in intracellular ROS levels. Also, minocycline, which neutralized the cytotoxic effects of rotenone, significantly increased intracellular ROS levels, thus providing further support for this argument. The mechanism by which rotenone decreased intracellular ROS levels in the present study is not clear.

Both the methanol and ethyl acetate extracts of *L. schweinfurthii* and *Z. capense* countered the decrease in intracellular ROS caused by rotenone exposure (Figure 2). Phytochemical analysis of *Z. capense* has revealed several bio-active compounds, including alkaloids, lignans, coumarins, amides, flavonoids and terpenes [38,49]. The coumarins, 7,8-dihydroxy-4-methyl coumarin (DHMC) and 7,8-diacetoxy-4-methyl coumarin (DAMC), and flavonoids quercetin and quercetin penta-acetate, have been shown to be potential oxidants [50]. The increases in intracellular ROS levels, compared to rotenone treatment alone, may be due to pro-oxidant effects exerted by these coumarins and flavonoids. *S. puniceus* and *C. bulbispermum* were also observed to inhibit the actions of rotenone on intracellular ROS levels (Figure 2). In fact, methanol extracts of both species actually increased intracellular ROS levels above that of the vehicle controls. As mentioned earlier, *S. puniceus* and *C. bulbispermum* are known to contain high alkaloid content. Alkaloids found in this family include heamanthin, distichamine (a.k.a. buphanidrine) and buphanamine, which are known to exert toxic effects [21]. It is possible that these alkaloids possess pro-oxidant effects resulting in the observed increased intracellular ROS levels.

#### Intracellular glutathione content

**Figure 3:**
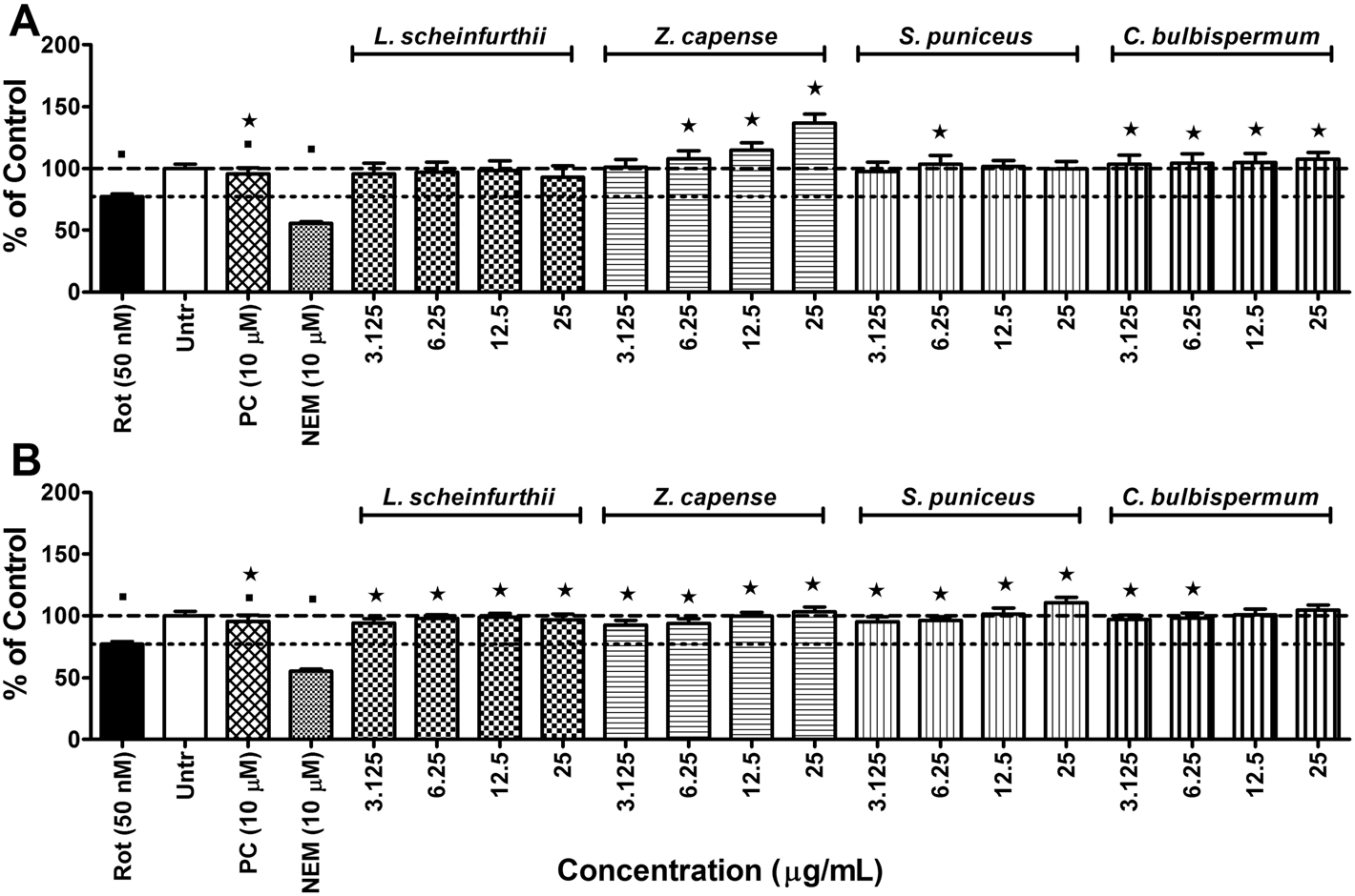
**Effects on intrinsic antioxidant capacity.** Intracellular glutathione content in SH-SY5Y cells exposed to rotenone at 50 nM after 1 h pre-treatment with either A) methanol or B) ethyl acetate plant extracts following 24 h exposure period. Significant differences from the rotenone control are indicated by* representing p value < 0.05, while significant differences from vehicle controls are indicated by ■, also representing p value < 0.05. PC = positive control (minocycline); Rot = rotenone; Untr = Vehicle controls. (The dashed-line indicates intracellular glutathione levels in the vehicle controls while the dotted-line is indicative of intracellular glutathione content in cells exposed to rotenone alone).

The positive control, NEM, depleted intracellular glutathione content, indicating that the assay produced expected results (Figure 3). Reduced intracellular glutathione content was also observed in cells treated with rotenone. This effect was counteracted by minocycline (Figure 3). As rotenone exposure did not produce any ROS generation, it is possible that it may have inhibited enzymes involved in glutathione synthesis or that it may have formed complexes with glutathione itself, decreasing the intracellular free reduced glutathione content. It is also possible that rotenone exposure could have caused the loss of intracellular glutathione by means of membrane leakage [51].

All of the plant extracts exerted effects similar to or greater than that of minocycline. Most extracts completely curbed the glutathione depletion induced by rotenone exposure (Figure 3A and B). The methanol extract of *Z. capense* had the greatest effect on intracellular glutathione content, producing significant increases in glutathione content in a dose-dependent manner (Figure 3A). Research has shown that quercetin limits the depletion of intracellular glutathione content by dehydroascorbic acid in red blood cells [52]. As *Z. capense* is known to contain quercetin, this may explain the effects of this plant extract observed in the present study. The highest test concentrations of the ethyl acetate extracts of both *S. puniceus* and *C. bulbispermum* also increased intracellular glutathione content above that of vehicle controls (Figure 3B). Results obtained with *C. bulbispermum* align with reports of other members of the *Crinum* species, where organic extracts curtailed glutathione depletion caused by CCl_4_ exposure in mice [53].

### Mitochondrial membrane potential

**Figure 4:**
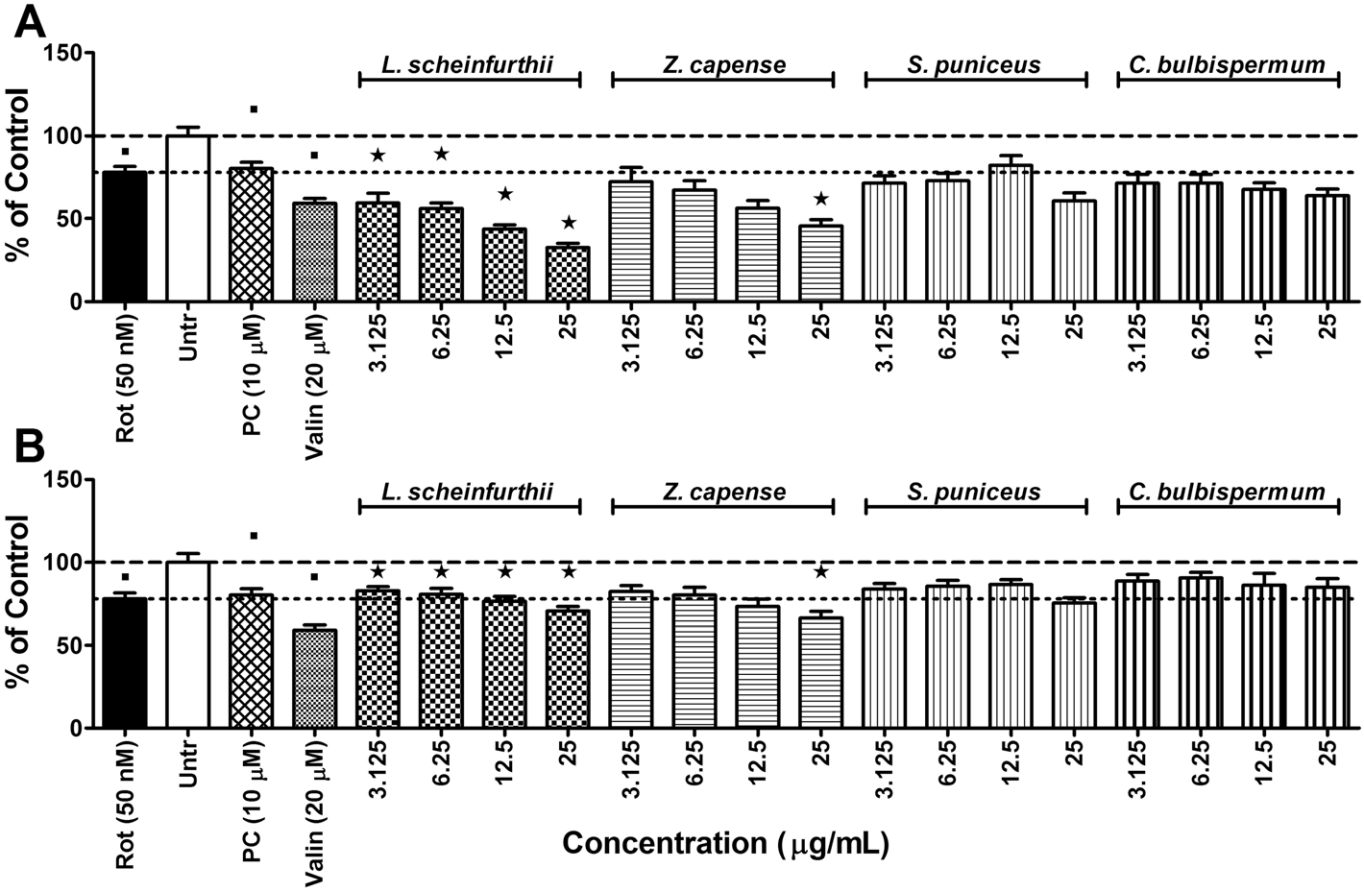
**Mitochondrial disturbances.** Mitochondrial membrane potential (MMP) in SH-SY5Y cells exposed to rotenone at 50 nM after 1 h pre-treatment with either A) methanol or B) ethyl acetate plant extracts following 24 h exposure period. Significant differences from the rotenone control are indicated by* representing p value < 0.05, while significant differences from vehicle controls are indicated by ■, also representing p value < 0.05. PC = positive control (minocycline at 10 μM); Rot = rotenone; Untr = Vehicle controls; Valin = valinomycin. (The dashed-line indicates MMP in the vehicle controls while the dotted-line is indicative of MMP levels in cells exposed to rotenone alone).

Valinomycin (20 μM), the assay positive control, caused a significant (*p <* 0.05) reduction in the MMP, compared to the vehicle controls, indicating that the assay produced expected results. Compared to vehicle controls, cells exposed to rotenone showed a significant (*p <* 0.05) reduction in the MMP (Figure 4). Minocycline pre-treatment did counteract the effect observed in cells exposed to rotenone, but the effect was small. Rotenone is known to inhibit the function of mitochondrial complex I, reducing ATP production from the electron transport chain (ETC) [48,51]. Mitochondrial uncoupling results in the irreversible formation of a large mitochondrial membrane permeability transition (MPT) pore on the inner mitochondrial membrane, which allows influx and efflux of ions and other large molecules as well as further dissipation of the MMP [51,54]. Therapeutic agents that can inhibit MPT pore formation would be beneficial in preventing MMP reduction and reduction in ATP production [55].

Generally, pre-treating cells with the methanol extracts of any one of the plants caused a further reduction in MMP, when compared to rotenone exposure alone (Figure 4A). Of the methanol extracts, pre-treatment with *L. schweinfurthii* demonstrated the greatest synergistic uncoupling effect, significantly (*p* < 0.05) reducing MMP beyond the effect of rotenone alone at all test concentrations. On the contrary, most test concentrations of the ethyl acetate extracts of the individual plants limited the degree to which rotenone treatment reduced MMP (Figure 4B). Of the ethyl acetate extracts, *C. bulbispermum* showed the greatest inhibition of the uncoupling effect of rotenone exposure.

Most of the tested extracts were not very effective in preventing the uncoupling effect of rotenone. This may be due to formation of the MPT pore, which is irreversible and has been reported to occur within 20 min of the rotenone apoptogenic effect, once mitochondrial dysfunction results [51]. Apart from this, MMP is not a static parameter and fluctuates with the respiratory needs of the cell. If a cell requires more energy (high ATP utilization) the MMP will decrease as ATP production increases. If less energy is required, the opposite will happen. It is therefore possible that the decreases in MMP caused by the plant extracts may be the result of increased ATP utilization and not necessarily mitochondrial uncoupling [56].

### Apoptosis

**Figure 5:**
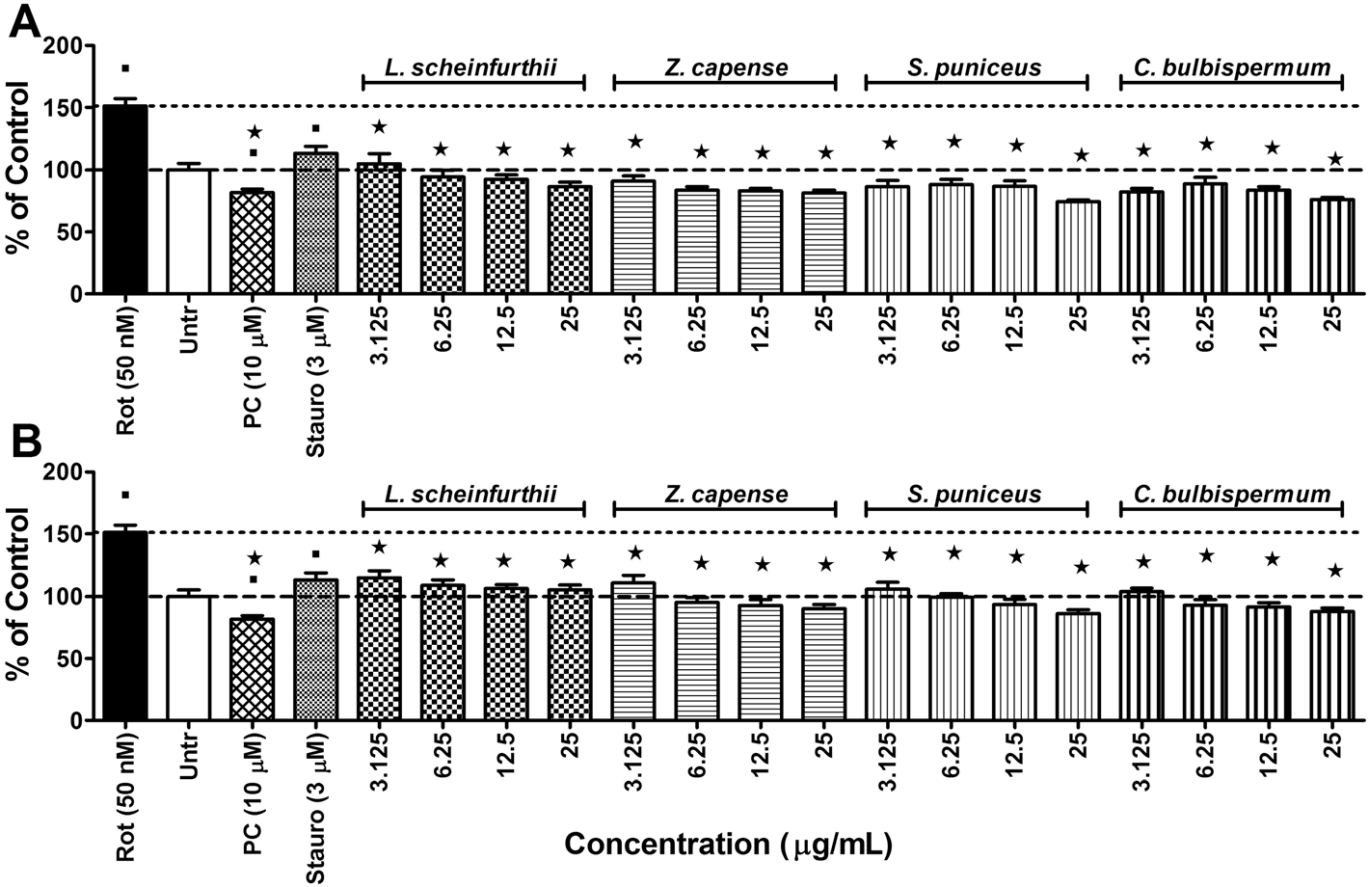
**Apoptotic cell death.** Caspase-3 activity in SH-SY5Y cells exposed to rotenone at 50 nM after 1 h pre-treatment with either A) methanol or B) ethyl acetate plant extracts following 24 h exposure period. Significant differences from the rotenone control are indicated by* representing p value < 0.05, while significant differences from vehicle controls are indicated by ■, also representing p value < 0.05. PC = positive control (minocycline at 10 μM); Rot = rotenone; Stauro = staurosporine; Untr = Vehicle controls. (The dashed-line indicates caspase-3 activity in the vehicle controls while the dotted-line is indicative of caspase-3 activity in cells exposed to rotenone alone).

Staurosporine, a general apoptosis inducer [57], caused a significant (*p <* 0.05) increase in caspase-3 activity, indicating that the assay produced expected results. Rotenone exposure caused a greater increase in caspase-3 activity than the positive control (Figure 5). This action was counteracted by minocycline. Rotenone-induced apoptosis is thought to occur as a consequence of mitochondrial dysfunction, which is triggered by many factors including: disruption in ATP production, MMP uncoupling, increased intracellular calcium levels, ROS generation and glutamate excitotoxicity, in neurons [51,58]. ATP depletion, due to a depolarized MMP, ultimately results in MPT pore formation [54]. The pore allows calcium influx into the cytosol, as well as the irreversible release of cytochrome C [51], causing formation of the apoptosome and subsequent caspase-3 activation. Minocycline has been reported to inhibit MPT pore formation thus preventing cytochrome C release resulting in suppression of apoptosis [59].

All plant extracts at all test concentrations were observed to significantly (*p <* 0.05) reduce rotenone-induced caspase-3 activity (Figure 5). Methanol extracts were more effective than ethyl acetate extracts. Both *S. puniceus* and *C. bulbispermum* methanol extracts (25 μg/ml), were more effective than minocycline at inhibiting rotenone-induced caspase-3 activation. Test concentrations of 6.25 - 25 μg/ml of the methanol extract of *Z. capense* resulted in a response comparable to that of minocycline (Figure 5A).

*Z. capense* contains the alkaloid, rutaecarpine [60], which has been shown to inhibit apoptosis in cardiocytes subjected to a hypoxia-reoxygenation cycle [61]. It is possible that this compound may also be responsible for suppressing caspase-3 activity in the present study. However, Bao *et al.*[61] proposed inhibition of NADPH oxidase-dependent ROS generation as the mechanism of action of rutaecarpine. As no ROS generation was observed in the present study this may disqualify rutaecarpine as the compound responsible for inhibiting rotenone-induced apoptosis in the present study. This needs to be confirmed through further experimentation. Quercetin is another bioactive compounds found in *Z. capense* that may have contributed to limiting the caspase-3 induction caused by rotenone exposure. A number of studies have reported a neuroprotective effect of quercetin, specifically related to inhibiting apoptosis in SH-SY5Y cells [62,63].

Interestingly, a number of compounds isolated from the methanol extract of *Z. capense* have been reported to actually induce apoptosis in cell cultures [60]. These include compounds like norchelerythrine [64] and episesamin [65]. The same is true for the Amaryllidaceae family, which is known to contain various pro-apoptotic alkaloids like crinamine [66] and distichamine [67]. It is possible that the concentrations of these individual compounds in the crude extracts were too low to induce apoptosis in the present study. Apart from this, differences in cell type may also contribute to any discrepancies.

## Conclusion

This is the first study to investigate the effects of *L. schweinfurthii*, *Z. capense*, *S. puniceus* and *C. bulbispermum* species on the studied parameters, using an *in vitro* PD-like model. These plant extracts limited the depletion of intracellular glutathione content caused by rotenone exposure and demonstrated potent anti-apoptotic effects, warranting further investigations into their neuroprotective potential.

## Abbreviations

AAPH: 2,2’-azobis-2-methyl-propanimide dihydrochloride; ATCC: American type culture collection; ATP: Adenosine triphosphate; CHAPS: 3-[(3-cholamidopropyl)dimethylammonio]-1propanesulfonate; DHMC: 7,8-dihydroxy-4-methyl coumarin; EDTA: Ethylenediaminetetraacetic acid; ETC.: Electron transport chain; FCS: Foetal calf serum; H2DCF-DA: 2’,7’-dichlorodihydrofluorescein diacetate; HEPES: 4-(2-hydoxyethyl)-1-piperazineethanesulfonic acid; JC-1: 5,5’,6,6’-tetrachloro-1,1’,3,3’-tetraethylbenzimidazolcarbocyanine iodide; MMP: Mitochondrial membrane potential; NEM: *N*-ethylmaleimide; PBS: Phosphate buffered saline; PD: Parkinson’s disease; ROS: Reactive oxygen species; PMSF: Phenylmethylsulfonyl fluoride; SEM: Standard error of the mean; SRB: Sulforhodamine B; TCA: Trichloroacetic acid.

## Competing interests

The authors declare that they have no competing interest.

## Authors’ contributions

VS: conception and design of study, interpretation of data, writing of the manuscript. KS: performed the experiments, collected and analysed the data, interpretation of data and wrote the first draft. JvT: constructed experimental designs, contributed in terms of data analyses and interpretation, and writing of the manuscript. All authors read and approved the final manuscript.

## Authors’ information

Department of Pharmacology, Faculty of Health Sciences, School of Medicine, University of Pretoria, Private Bag X323, Arcadia 0007, South Africa.

## Pre-publication history

The pre-publication history for this paper can be accessed here:

http://www.biomedcentral.com/1472-6882/13/353/prepub

## References

[B1] Thomas B Beal MF Parkinson’s disease Hum Mol Genet 2007 13 183 194 10.1093/hmg/ddm159 17911161

[B2] Van Den Eeden SK Tanner CM Bernstein AL Leimpeter A Bloch DA Nelson LM Incidence of Parkinson’s disease: variation by age, gender and race/ethnicity Am J Epidemiol 2003 13 1015 1022 10.1093/aje/kwg068 12777365

[B3] de Lau LM Breteler MM Epidemiology of Parkinson’s disease Lancet Neurol 2006 13 25 35 10.1016/S1474-4422(05)70274-X 16713924

[B4] Maguire-Zeiss KA Federoff HJ Convergent pathobiologic model of Parkinson’s disease Ann NY Acad Sci 2003 13 152 166 12846984 10.1111/j.1749-6632.2003.tb07473.x

[B5] Joshi S Guleria R Pan J DiPette D Singh US Retinoic acid receptors and tissue-transglutaminase mediate short-term effect of retinoic acid on migration and invasion of neuroblastoma SH-SY5Y cells Oncogene 2006 13 240 247 16158052 10.1038/sj.onc.1209027

[B6] Ciccarone V Spengler BA Meyers MB Biedler JL Ross RA Phenotypic diversification in human neuroblastoma cells: expression of distinct neural crests lineages Cancer Res 1989 13 219 225 2535691

[B7] Höglinger GU Carrard G Michel PP Medja F Lombès A Ruberg M Friguet B Hirsch EC Dysfunction of mitochondrial complex I and the proteasome: interactions between two biochemical deficits in a cellular model of Parkinson’s disease J Neurochem 2003 13 1297 1307 10.1046/j.1471-4159.2003.01952.x 12911637

[B8] Ruipérez V Darios F Davletov B Alpha-synuclein, lipids and Parkinson’s disease Prog Lipid Res 2010 13 420 428 10.1016/j.plipres.2010.05.004 20580911

[B9] Sherer TB Betarbet R Testa CM Seo BB Richardson JR Kim JH Miller GW Yagi T Matsuno-Yagi A JT G Mechanism of toxicity in rotenone models of Parkinson’s disease J Neurosci 2003 13 10756 10764 14645467 10.1523/JNEUROSCI.23-34-10756.2003 PMC6740985

[B10] Schuler F Casida JE Functional coupling of PSST and ND1 subunits in NADH: ubiquinone oxidoreductase established by photoaffinity labeling Biochim Biophys Acta 2001 13 79 87 10.1016/S0005-2728(01)00183-9 11418099

[B11] Hu BY Weick JP Yu J Ma LX Zhang XQ Thomson JA Zhang SC Neural differentiation of human induced pluripotent stem cells follows developmental principles but with variable potency Proc Natl Acad Sci U S A 2010 13 4335 4340 10.1073/pnas.0910012107 20160098 PMC2840097

[B12] Hu S Han R Mak S Han Y Protection against 1-methyl-4-phentlpyridinium ion (MPP+)-induced apoptosis by water extract of ginseng (Panax ginseng C.A Meyer) in SH-SY5Y cells J Ethnopharmacol 2011 13 34 42 10.1016/j.jep.2011.02.017 21349320

[B13] Skibinski G Finkbeiner S Drug discovery in Parkinson’s disease: update and developments in the use of cellular models Int J High Throughput Screen 2011 13 15 25 23505333 10.2147/IJHTS.S8681 PMC3596173

[B14] Chung V Liu L Bian Z Zhao Z Leuk FW Kum WF Gao J Li M Efficacy and safety of herbal medicines for idiopathic Parkinson’s disease: a systematic review Mov Disord 2006 13 1709 1715 10.1002/mds.21008 16830309

[B15] Martorana A Esposito Z Koch G Beyond the cholinergic hypothesis: do current drugs work in alzheimer’s disease? CNS Neurosci Ther 2010 13 235 245 20560995 10.1111/j.1755-5949.2010.00175.x PMC6493875

[B16] Kim J Wong PKY Oxidative stress is linked to ERK1/2-p16 signalling-mediated growth defect in ATM-deficient astrocytes J Biol Chem 2009 13 14396 14404 10.1074/jbc.M808116200 19321450 PMC2682888

[B17] Doo AR Kim SN Park JY Cho KH Hong J Eun-Kyung K Moon SK Jung WS Lee H Jung JH Park HJ Neuroprotective effects of an herbal medicine, Yi-Gan San on MPP+/ MPTP-induced cytotoxicity in vitro and in vivo J Ethnopharmacol 2010 13 433 442 10.1016/j.jep.2010.07.008 20633628

[B18] Ferry P Johnson M Wallis P Use of complementary therapies and non-prescribed medication in patients with Parkinson’s disease Postgrad Med J 2002 13 612 614 10.1136/pmj.78.924.612 12415085 PMC1742510

[B19] Mabogo DEN The ethnobotany of the VhaVenda 1990 Pretoria, South Africa: MSc thesis, University of Pretoria

[B20] Van Wyk B-E Gericke N People’s plants: a guide to useful plants of southern Africa 2000 Pretoria: Briza Publications

[B21] Watt JM Breyer-Brandwijk MG The medicinal and poisonous plants of Southern and Eastern Africa 1962 2 London: Livingstone

[B22] Veale DJH Furman KI Oliver DW South African traditional herbal medicines used during pregnancy and child-birth J Ethnopharmacol 1992 13 185 191 10.1016/0378-8741(92)90043-Q 1434676

[B23] Oloyede GK Oke JM Raji Y Olugbade TA Antioxidant and anticonvulsant alkaloids in *Crinum ornatum* bulb extract World J Chem 2010 13 26 31

[B24] Vichai V Kirtikara K Sulforhodamine B Colorimetric assay for cytotoxicity screening Nat Protoc 2006 13 1112 1116 10.1038/nprot.2006.179 17406391

[B25] Faust K Gehrke S Yang Y Yang L Beal MF Lu B Neuroprotective effects of compounds with antioxidant and anti-inflammatory properties in a Drosophilia model of Parkinson’s disease BMC Neurosci 2009 13 1 17 10.1186/1471-2202-10-1 19723328 PMC3152779

[B26] Shaykhalishahi H Yazdanparast R Ha HH Chang YT Inhibition of H2O2-induced neuroblastoma cell cytotoxicity by a triazine derivative, AA3E2 Eur J Pharmacol 2009 13 1 6 10.1016/j.ejphar.2009.07.017 19619524

[B27] Nair JJ Rárová L Strnad M Bastida J van Staden J Apoptosis-inducing effects of distichamine and narciprimine, rare alkaloids of the plant family Amaryllidaceae Bioorg Med Chem Lett 2012 13 6195 6199 10.1016/j.bmcl.2012.08.005 22921081

[B28] Sternfeld T Schmid M Tischleder A Mudra S Schlamp A Kost BP Gruber R Youle M Bogner JR Goebel FD The influence of HIV infection and antiretroviral therapy on the mitochondrial membrane potential of peripheral mononuclear cells Antiviral Ther 2007 13 769 778 17713160

[B29] van Tonder JJ Development of an in vitro mechanistic toxicity screening model using cultured hepatocytes 2012 Pretoria: PhD dissertation, University of Pretoria

[B30] Gilgun-Sherki Y Melamed E Offen D Oxidative stress induced-neurodegenerative diseases: the need for antioxidants that penetrate the blood brain barrier Neuropharmacology 2001 13 959 975 10.1016/S0028-3908(01)00019-3 11406187

[B31] Chauhan V Chauhan A Oxidative stress in Alzheimer’s disease Pathophysiology 2006 13 195 208 10.1016/j.pathophys.2006.05.004 16781128

[B32] Jordan J Cena V Prehn JH Mitochondrial control of neuron death and its role in neurodegenerative disorders J Physiol Biochem 2003 13 129 141 10.1007/BF03179878 14649878

[B33] Louvena N Cohen JW Han LY Talbot K Wilson RS Bennett DA Trojanowski JQ Arnold SE Caspase-3 is enriched in postsynaptic densities and increased in Alzheimer’s disease Am J Pathol 2008 13 1488 1495 10.2353/ajpath.2008.080434 18818379 PMC2570138

[B34] Tatton WG Chalmers-Redman R Brown D Tatton N Apoptosis in Parkinson’s disease: signals for neuronal degradation Ann Neurol 2003 13 61 70 10.1002/ana.10489 12666099

[B35] Betarbet R Sherer TB MacKenzie G Garcia-Osuna A Panov V Greemamyre JT hronic systemic pesticide exposure reproduces features of Parkinson’s diseases Nat Neurosci 2000 13 1301 1306 10.1038/81834 11100151

[B36] Swarnkar S Goswami P Kamat PK Gupta S Patro IK Singh S Nath C Rotenone-induced apoptosis and role of calcium: a study on Neuro-2a cells Arch Toxicol 2012 13 1387 1397 10.1007/s00204-012-0853-z 22526376

[B37] Moshi MJ Kamuhabwa A Mbwambo Z de Witte P Cytotoxic screening of some Tanzania medicinal plants East Central Afr J Pharmaceutical Sci 2003 13 52 56

[B38] Patiño LOJ Prieto RJ Cuca SLE Rasooli I Zanthoxylum genus as potential source of bioactive compounds Bioactive Compounds in Phytomedicine 2012 Rijeka, Croatia: InTech 185 218

[B39] Bastida J Lavilla R Viladomat F Chemical and biological aspects of Narcissus alkaloids Alkaloids Chem Biol 2006 13 87 179 17133715 10.1016/S1099-4831(06)63003-4 PMC7118783

[B40] Klein NC Cunha BA Tetracyclines Med Clin North Am 1995 13 789 801 7791423 10.1016/s0025-7125(16)30039-6

[B41] Wu DC Jackson-Lewis V Vila M Tieu K Teismann P Vadseth C Choi DK Ischiropoulos H Przedborski Blockade of microglial activation is neuroprotective in the 1-methyl-4-phenyl-1,2,3,6-tetrahydropyridine mouse model of Parkinson disease J Neurosci 2002 13 1763 1771 11880505 10.1523/JNEUROSCI.22-05-01763.2002 PMC6758858

[B42] Kremlev SG Roberts RL Palmer C Differential expression of chemokines and chemokine receptors during microglial activation and inhibition J Neuroimmunol 2004 13 1 9 10.1016/j.jneuroim.2003.11.012 15020059

[B43] Keeney PM Xie J Capaldi RA Bennett JP Parkinson’s disease brain mitochondrial complex I has oxidatively damaged subunits and is functionally impaired and misassembled J Neurosci 2006 13 5256 5264 10.1523/JNEUROSCI.0984-06.2006 16687518 PMC6674236

[B44] Perier C Tieu C Guégan C Caspersen C Jackson-Lewis V Carelli V Martinuzzi A Hirano M Przedborski S Vila M Complex I deficiency primes Bax-dependent neuronal apoptosis through mitochondrial oxidative damage Proc Natl Acad Sci USA 2005 13 19126 19131 10.1073/pnas.0508215102 16365298 PMC1323177

[B45] Molina-Jimenez MF Sanchez-Reus MI Benedi J Effect of fraxetin and myricetin on rotenone-induced cytotoxicity in SH-SY5Y cells: comparison with N-acetylcysteine Eur J Pharmacol 2003 13 81 87 10.1016/S0014-2999(03)01902-2 12860476

[B46] Vrablic AS Albright CD Craciunescu CN Salganik RI Zeisel SH Altered mitochondrial function and overgeneration of reactive oxygen species precede the induction of apoptosis by 1-Ο-octadecyl-2-methyl-rac-glycero-3-phosphocholine in p53-defective hepatocytes FASEB J 2001 13 1739 1744 10.1096/fj.00-0300com 11481221

[B47] Gao HM Liu B Hong JS Critical role for microglial NADPH oxidase in rotenone-induced degeneration of dopaminergic neurons J Neurosci 2003 13 6181 6187 12867501 10.1523/JNEUROSCI.23-15-06181.2003 PMC6740554

[B48] Li J Spletter ML Johnson DA Svendsen CN Johnson JA Rotenone-induced caspase 9/3-independent and -dependent cell death in undifferentiated human neural stem cells J Neurochem 2005 13 462 476 10.1111/j.1471-4159.2004.02872.x 15659217

[B49] Adesina SK The Nigerian Zanthoxylum: chemical and biological values AJTCAM 2005 13 282 301

[B50] Koshy AS Anila L Vijayalaksmi NR Flavonoids from *Garciniacombagia* lower lipid levels in hypercholesterlemic rats Food Chem 2001 13 289 294 10.1016/S0308-8146(00)00225-9

[B51] Isenberg JS Klaunig JE Role of mitochondrial membrane permeability transition (MPT) in rotenone-induced apoptosis in liver cells Toxicol Sci 2000 13 340 351 10.1093/toxsci/53.2.340 10696782

[B52] Fiorani M de Sanctis R Menghinello P Cucchiarini L Cellini B Dachà M Quercetin prevents glutathione depletion induced by dehydroascorbic acid in rabbit red blood cells Free Radic Res 2001 13 639 648 10.1080/10715760100300531 11697039

[B53] Ilavenil S Hepatoprotective mechanism of Crinum asiaticum L. and lycorine in carbon tetrachloride induced oxidative stress in Swiss albino mice 2012 Thanjavur, India: PhD thesis, Prist University

[B54] Kroemer G Petit PX Zamzami N Vayssiere JL Mignotte B The biochemistry of programmed cell death FASEB J 1995 13 1277 1287 7557017 10.1096/fasebj.9.13.7557017

[B55] Zamzami N Susin SA Marchetti P Hirsch T Castedo M Kroemer G Mitochondrial control of nuclear apoptosis J Exp Med 1996 13 1533 1544 10.1084/jem.183.4.1533 8666911 PMC2192517

[B56] Chandel NS Budinger GR Choe SH Schumacker PT Cellular respiration during hypoxia. Role of cytochrome oxidase as the oxygen sensor in hepatocytes J Biol Chem 1997 13 18808 18816 10.1074/jbc.272.30.18808 9228055

[B57] Thuret G Chiquet C Herrag S Dumollard J-M Boudard D Bednarz J Campos L Gain P Mechanisms of staurosporine induced apoptosis in a human corneal endothelial cell line Brit J Ophthalmol 2003 13 346 352 10.1136/bjo.87.3.346 12598452 PMC1771564

[B58] Chauvin C De Oliveira F Ronot X Mousseau M Leverve X Fontaine E Rotenone inhibits the mitochondrial permeability transition-induced cell death in U937 and KB cells J Biol Chem 2001 13 41394 41398 10.1074/jbc.M106417200 11527970

[B59] Zhu S Stavrovskaya IG Drozda M Kim BY Ona V Li M Sarang S Liu AS Hartley DM Wu DC Gullans S Ferrante RJ Przedborski S Kristal BS Friedlander RM Minocycline inhibits cytochrome c release and delays progression of amyotrophic lateral sclerosis in mice Nature 2002 13 74 78 10.1038/417074a 11986668

[B60] Mansoor TA Borralho PM Luo X Mulhovo S Rodrigues CM Ferreira MJ Apoptosis inducing activity of benzophenanthridine-type alkaloids and 2-arylbenzofuran neolignans in HCT116 colon carcinoma cells Phytomedicine 2013 13 923 929 23643093 10.1016/j.phymed.2013.03.026

[B61] Bao MH Dai W YJ L Hu CP Rutaecarpine prevents hypoxia-reoxygenation-induced myocardial cell apoptosis via inhibition of NADPH oxidases Can J Physiol Pharmacol 2011 13 177 186 10.1139/Y11-006 21423291

[B62] Suematsu N Hosoda M Fujimori K Protective effects of quercetin against hydrogen peroxide-induced apoptosis in human neuronal SH-SY5Y cells Neurosci Lett 2011 13 223 227 10.1016/j.neulet.2011.09.028 21964380

[B63] Xi J Zhang B Luo F Liu J Yang T Quercetin protects neuroblastoma SH-SY5Y cells against oxidative stress by inhibiting expression of Krüppel-like factor 4 Neurosci Lett 2012 13 115 120 10.1016/j.neulet.2012.08.082 22985515

[B64] Yamamoto S Seta K Morisco C Vatner SF Sadoshima J Chelerythrine rapidly induces apoptosis through generation of reactive oxygen species in cardiac myocytes J Mol Cell Cardiol 2001 13 1829 1848 10.1006/jmcc.2001.1446 11603925

[B65] Miyahara Y Komiya T Katsuzaki H Imai K Nakagawa M Ishi Y Hibasami H Sesamin and episesamin induce apoptosis in human lymphoid leukemia Molt 4B cells Int J Mol Med 2000 13 43 46 10851264

[B66] McNulty J Nair JJ Codina C Bastida J Pandey S Gerasimoff J Griffin C Selective apoptosis-inducing activity of crinum-type Amaryllidaceae alkaloids Phytochemistry 2009 13 1068 1074 10.1016/j.phytochem.2007.01.006 17331551

[B67] Nair S Singh SV Krishan A Flow cytometric monitoring of glutathione content and anthracycline retention in tumor cells Cytometry 1991 13 336 342 10.1002/cyto.990120408 1648468

